# Interpenetrating Polymer Network Based on Polyether-Polyester Polyurethane and Epoxy Resin

**DOI:** 10.3390/polym18020209

**Published:** 2026-01-12

**Authors:** Chao Feng, Zhiqiang Song, Dongdong Xu, Fei Wan, Andreas Hermann Gerdes, Lan Wang, Linlin Zhang

**Affiliations:** 1 School of Civil Engineering, Qingdao University of Technology, Qingdao 266033, China; 2 Institute of Functional Interfaces (IFG), Karlsruhe Institute of Technology (KIT), 76344 Karlsruhe, Germany

**Keywords:** polyurethane (PU), epoxy resin (EP), prepolymer, interpenetrating network structure (IPNs)

## Abstract

Epoxy resins often require toughening to broaden their engineering applications, such as in durable concrete repair. This study addresses this need by developing high-performance polyurethane/epoxy (PU/EP) interpenetrating polymer networks (IPNs). The composites were synthesized via prepolymer and stepwise methods using polyether polyol (PPG-1000), isocyanate (MDI-50), and E51 epoxy. At an optimal PU prepolymer content of 15 wt%, the polyether-based IPNs achieved a balanced mechanical profile (tensile strength: 59.90 MPa; elongation at break: 6.46%; compressive strength: 69.99 MPa). Further tuning of the soft segment by introducing polyester polyol (PS-2412) yielded superior performance at a PS-2412/PPG-1000 ratio of 30/70. This formulation increased tensile and compressive strengths by 11.4% and 6.07% (to 66.74 MPa and 74.24 MPa), and dry and wet bond strengths by 12.1% and 36.3% (to 5.68 MPa and 4.62 MPa), respectively. The enhancement is attributed to the increased crosslinking density and more uniform network structure imparted by PS-2412, which improves stress distribution and interfacial adhesion. This work provides an effective soft-segment design strategy for fabricating toughened epoxy composites with robust mechanical and adhesive properties.

## 1. Introduction

Epoxy resins were widely used in engineering due to their high stiffness [1], strong creep resistance [2,3], and chemical stability [4]. However, their inherent brittleness limited their application in many demanding scenarios. To overcome this, various toughening strategies had been developed, among which the construction of interpenetrating polymer networks (IPNs) had proven particularly effective [5,6,7]. IPNs were characterized by the permanent entanglement of two or more polymer networks, typically leading to synergistic improvements in mechanical properties [8,9,10].

Polyurethane (PU) was one of the most commonly used polymers for toughening epoxy resins. Its segmental designability allowed for precise adjustment of compatibility and performance in PU/epoxy (PU/EP) IPNs [11]. Polyether-based PUs offered good low-temperature flexibility and hydrolysis resistance [12], while polyester-based PUs contributed higher mechanical strength and thermal stability [13]. However, simple blending of these two types often suffered from poor compatibility, which restricted property integration.

Recent studies demonstrated the potential of PU/EP IPNs. For example, Zhang et al. [14] used epoxy-terminated polyurethane to modify epoxy, achieving increases of 67.5% in elongation at break and 28.3% in toughness at 10 phr loading. Li et al. [11] reported a waterborne PU/EP system where a mixed polyol approach (polyether/polyester) enhanced tensile strength and elongation by over 100%. Similarly, ROUDSARI et al. [15] found that adding polypropylene carbonate polyol significantly improved impact and tensile strength. In other work, Li et al. [16] developed a PU/EP IPN with a 50/50 ratio that exhibited a tensile strength of 13.9 MPa, an elongation at break of 274.1%, and additional functionalities such as self-healing and shape memory. WU et al. [17] used two hydroxyl-terminated polyurethanes, HTPU1 and HTPU2 (with different ratios of polyether and polyester), as epoxy modifiers to study the effects of HTPU on the material system at room temperature and 77 K. At a content of 10 phr for HTPU1, the impact strength, tensile strength, and elongation at break of the EP/HTPU1 by 77%, 8%, and 40% higher than those of the control group at room temperature. At 77 K, the impact strength of EP/HTPU1 did not significantly increase until the content of HTPU1 reached 20 phr. The trend in impact strength for HTPU2 was similar to that of HTPU1 at room temperature. At both room temperature and 77 K, the highest impact strength of EP/HTPU2 at 10 phr of HTPU2 was 35.8 kJ/m^2^. Despite these advances, achieving a balanced combination of high strength, good toughness, and strong adhesion—particularly for demanding applications such as concrete repair—remained a challenge, especially when compatibility between different polyols was limited.

To address this, the present study introduced a compatible phthalic anhydride-based polyester polyol (PS-2412) as a blending soft segment with polyether polyol (PPG-1000). We synthesized a series of isocyanate-terminated polyurethane prepolymers with varying PS-2412/PPG-1000 ratios and incorporated them into epoxy to form PU/EP IPNs. Unlike previous studies that often focused on a single property or used poorly compatible polyol blends, our work systematically tailored the soft-segment chemistry to simultaneously enhance tensile strength, compressive strength, dry/wet bond strength, and thermal stability, specifically targeting high-performance concrete repair applications. The novelty lay in the use of a highly compatible polyester/polyether hybrid soft segment to create a homogeneous, densely crosslinked IPN structure that overcame the typical trade-offs between strength, toughness, and adhesion.

## 2. Experiment

### 2.1. Materials

Methylene diphenyl diisocyanate (MDI-50) (Sigma-Aldrich, St. Louis, MO, USA), Polypropylene glycol (PPG-1000) (Sigma-Aldrich, St. Louis, USA). Methylbenzene (Sigma-Aldrich, St. Louis, USA). Bromocresol green (Sigma-Aldrich, St. Louis, USA). Isopropanol (Sigma-Aldrich, St. Louis, USA). Dibutylamine (Sigma-Aldrich, St. Louis, USA). Epoxy resin E51 (Baichen Insulation Material, Laizhou, China). Diethylene glycol phthalic anhydride polymer (PS-2412) (Renaud Chemical, Shanghai, China). Low-molecular-weight polyamide 651 (Xinsu New Material, Suzhou, China). Polyetheramine D230 (Sigma-Aldrich, St. Louis, USA). The curing agent used in the experiment was a mixture of low-molecular-weight polyamide 651 and polyetheramine D230 in a 2:3 ratio. The ordinary Portland cement used was sourced from Qingdao Shanshui Hengtai Cement Co., Ltd. (Qingdao, China).

### 2.2. Matching Ratio

Based on the principle of polypropylene glycol, diethylene glycol phthalic anhydride polymer (PS-2412) was used in various proportions to blend with polyether polyol (PPG-1000) to prepare polyurethane prepolymers. The PS-2412 and PPG-1000 blending ratios were divided into six groups, as shown in Table 1.

### 2.3. ω(NCO%) Theoretical Calculation and Determination

Based on preliminary experiments and an extensive literature review [18,19], the ω(NCO%) for synthesizing polyurethane prepolymers was 10%. The theoretical calculation formula for ω(NCO%) is given in Equation (1).

(1) $$\omega (\mathrm{NCO}\%)=\frac{\left(\frac{m_{\mathrm{NCO}}\;\times \;w_{\mathrm{NCO}}}{42}-\frac{m_{\mathrm{OH}}}{\frac{M_{\mathrm{OH}}}{n}}\right)\times 42}{m_{\mathrm{NCO}}+m_{\mathrm{OH}}}$$

*m*_NCO_ is the added mass of the isocyanate; *w*_NCO_ is the percentage of -NCO in the isocyanate (NCO%); 42 is the molar mass of the isocyanate group; *m*_OH_ is the added mass of the polyol; *M*_OH_ is the molecular weight of the polyol; n is the number of polyol hydroxyl functional groups.

### 2.4. Polyurethane/Epoxy Composite Material Performance Analysis

#### 2.4.1. Determination of Isocyanate Group Content

A total of 1.00 g of the sample was weighed into a dry conical flask, 25.00 mL of methylbenzene was added to dissolve the sample, 25.00 mL of dibutylamine methylbenzene solution was added, sealed with a stopper, shaken thoroughly, and let stand for 15 min. A volume of 100 mL of isopropanol and five drops of bromocresol green indicator were added, and then titrated with 0.1 mol/L HCl standard solution to the endpoint (from blue to yellow). Blank experiments were simultaneously conducted. The blank experiment was conducted under the same reagent quantities and reaction conditions as the sample experiment, except that no sample was added. The formula for calculating the -NCO content is given in Equation (2).

(2) $$\omega \left(\mathrm{NCO}\%\right)=\frac{\left(V-V^{0}\right)\times c\times 42}{m}\times 100\%$$

*V* is volume of hydrochloric acid standard solution consumed by the sample group (mL); *V*^0^ is volume of hydrochloric acid standard solution consumed by the blank control group (mL); *c* is concentration of hydrochloric acid standard solution (0.1 mol/L); *m* is mass of polyurethane prepolymer (g); ω(NCO%) is content of isocyanate groups in the polyurethane prepolymer (%).

#### 2.4.2. Fourier Transform Infrared (FTIR)

The German Bruker (Karlsruhe, Germany) company’s VERTEX80 type Fourier transform infrared (FT-IR) spectrometer was used to probe the vibration of chemical molecules. The range is 4000–500 cm^−1^, with 32 scans and 4 cm^−1^ resolution.

#### 2.4.3. Thermogravimetric Analysis (TGA)

The Beijing Hengjiu Experimental Equipment Co., Ltd. (Beijing, China). STA-7000 simultaneous thermal analyzer was used to test the thermal stability properties of the samples. The temperature rose from 50 to 600 °C at 3 °C/min.

#### 2.4.4. Scanning Electron Microscopy (SEM) Characterization

The German ZEISS (Jena, Germany) company’s JSM-6400 scanning electron microscope was used to observe the surface morphology of the sample.

#### 2.4.5. Tensile and Compression Tests

The tensile strength, elongation at break, and compressive strength of the specimens were tested in accordance with GB/T 2567-2021 [20], using a WDS-100000 universal mechanical testing machine Jinan zhongluchang Testing Machine Manufacturing Co., Ltd., Jinan, China). The dimensions of the samples used for testing tensile strength and elongation at break were as follows: length 200 mm, width 20 mm, thickness 4 ± 0.2 mm, and gauge length 50 mm. The dimensions of the samples used for testing compressive strength were 100 mm × 100 mm × 200 mm.

#### 2.4.6. Bond Strength Tests

**Figure 1:**
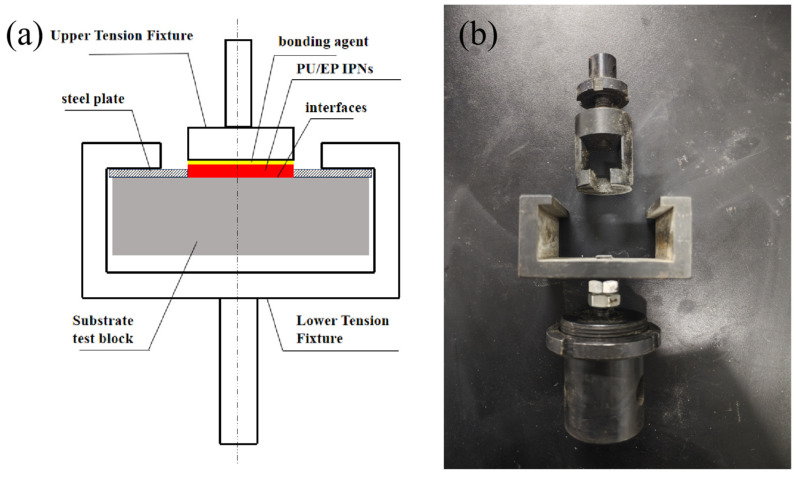
(**a**) Schematic diagram of base sample size and bonding strength test device, and (**b**) physical drawing of test device.

Mortar specimens of 70 mm × 70 mm × 20 mm were prepared, and repair materials were applied with the coating thickness controlled between 0.5 mm and 1 mm. The test area was 40 mm × 40 mm. The WDS-100,000 mechanical testing machine was used to test the bond strength. The bonding performance was evaluated in accordance with GB/T 16777-2008 [21]. After the pull-out blocks were bonded to the coating surface using a high-strength adhesive, tensile loads were applied to test the dry bond strength and wet bond strength. The experimental setup is shown in Figure 1.

#### 2.4.7. Concrete Repair Performance Tests

##### Adhesive Compressive and Flexural Strength Tests

The tests were conducted in accordance with JTG3420-2020 [22]. Cement mortar specimens were prepared in two geometries: (1) 70.7 mm × 70.7 mm × 70.7 mm cubes for compressive strength tests, and (2) 40 mm × 40 mm × 160 mm prisms for flexural strength tests. The WDS-100,000 mechanical testing machine was used to conduct splitting tensile tests and flexural strength tests on the cement blocks. After failure, the IPN repair material was evenly applied to the fracture surface of the specimens. After the specimens were destroyed, the IPN repair material was evenly applied to the fracture surface, the specimens were bonded, and then placed in a curing tank at a temperature of 20 ± 3 °C and a relative humidity of 50%~70% for 72 h of curing. Then, the splitting tensile strength tests and flexural strength tests were performed again.

##### Antifreeze Performance Tests

The TDR-II freeze–thaw cycle box was used for frost resistance testing. All the aforementioned bonded compression and bonded flexural test specimens underwent freeze–thaw cycle tests using the slow freezing method in accordance with GB/T 50082-2009 [23]. The freeze–thaw cycles were conducted with a high temperature of (5 ± 2)°C and a low temperature of (−10 ± 2)°C. Each complete cycle was completed within 3 to 5 h. After every 25 cycles (for a total of 100 cycles), the specimens were removed. Their surfaces were cleaned, and the morphological changes at the repair interface were observed. Subsequently, mechanical property tests were performed on them, and the failure modes were recorded.

### 2.5. Polyurethane/Epoxy Composite Material Preparation

#### 2.5.1. Experimental Principle

**Figure 2:**
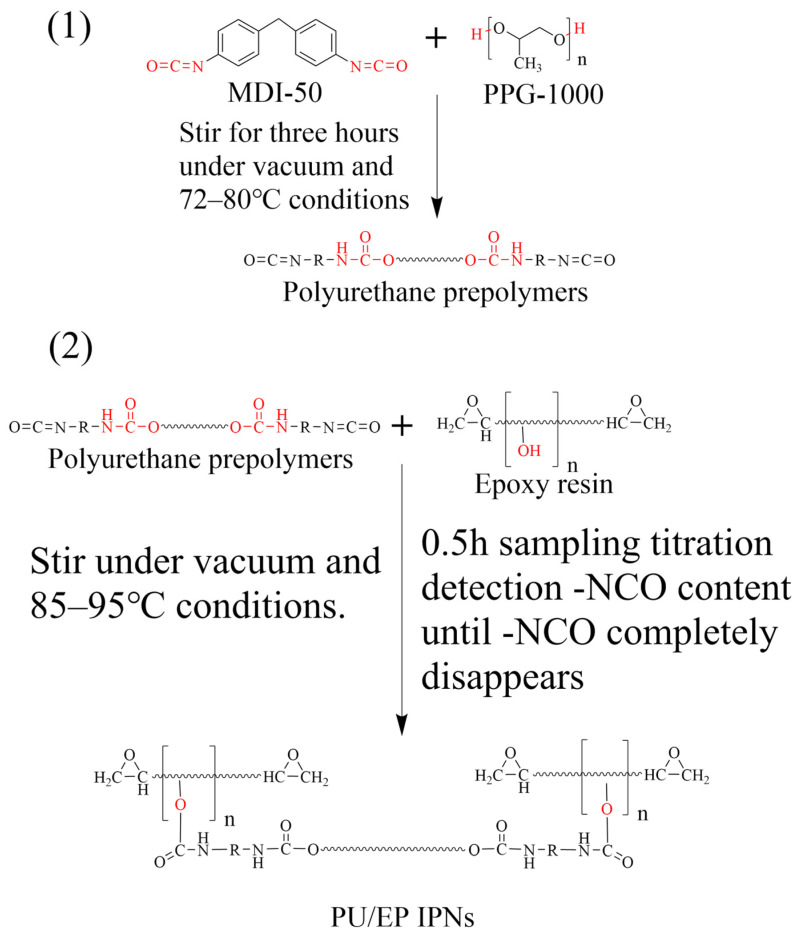
Flow chart of the reaction process of PU-prepolymer and PU/EP IPNs.

Using the strong designability of the polyurethane (PU) molecular structure and the presence of hydroxyl groups in the diepoxy novolac (DGEBA) epoxy resin, the terminal isocyanate groups of the PU prepolymer can be reacted with the pendent hydroxyl groups of the epoxy resin through a chemical-stoichiometric high-temperature reaction to synthesize a polyurethane/epoxy resin amorphous interpenetrating polymer network structure (PU/EP IPNs). The successful grafting of the PU prepolymer can effectively improve the fracture toughness of the epoxy resin, thereby achieving the purpose of toughening and modifying the epoxy resin [14]. The reaction process is shown in Figure 2.

#### 2.5.2. Preparation of Polyether-Based Polyurethane/Epoxy Resin Composites

##### Pre-Treatment of Raw Materials

The calculated amounts of polypropylene glycol PPG-1000) and epoxy resin (E51) were weighed separately and placed in a vacuum drying oven. The raw material was heated under vacuum at 120 °C for 3 h to remove excess moisture from the raw materials (<0.05%). The materials were sealed and allowed to cool to room temperature for later use.

##### Synthesis of PU-Prepolymer

A circulating oil bath was preheated to a constant temperature of 50 °C. Added a certain amount of pre-treated MDI-50 and PPG-1000 to a three-necked flask. The flask was placed in the circulating oil bath equipped with a stirring device and connected to a circulating water vacuum pump. It was stirred under vacuum at 120 r/min, and heating continued to a reaction temperature of 72–80 °C for 3 h to obtain the polyurethane prepolymer.

##### Preparation of PU/EP IPNs

An appropriate amount of pre-treated epoxy resin E51 was weighed into a three-necked flask, and different mass fractions of polyurethane prepolymer were added. The reaction setup was the same as described above. It was stirred under vacuum at 85–95 °C and sampled every 0.5 h to titrate and detect the -NCO content until the -NCO completely disappeared, indicating that the reaction is complete. The product was poured into an epoxy resin curing agent, mixed thoroughly, and placed in a vacuum drying oven for degassing. Then, it was poured into a mold for shaping, and after heating in a convection oven at 80 °C for 6 h, it was demolded (or cured at room temperature for 24 h) to obtain the PU/EP IPNs. The PU prepolymer content was prepared at 0%, 5%, 10%, 15%, 20%, and 25%, named PU-0%, PU-5%, PU-10%, PU-15%, PU-20%, and PU-25%, respectively.

#### 2.5.3. Preparation of Polyether-Polyester-Based Polyurethane/Epoxy Resin Composites

**Figure 3:**
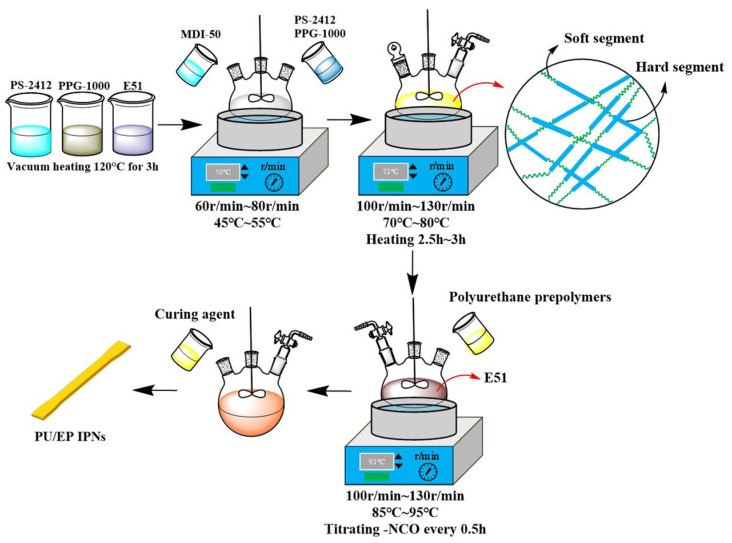
Schematic diagram of the preparation processes for the polyurethane prepolymer and the PU/EP IPNs.

During the synthesis of prepolymers, PS-2412 and PPG-1000 were added in different ratios, and the remaining steps were consistent with the above experiment. The samples were PU0, PU10, PU20, PU30, PU40, and PU50. The preparation process is illustrated in Figure 3.

### 2.6. Preparation of Cementitious Specimens

The test specimens were prepared in accordance with JTG3420-2020 [22]. Cement mortar specimens were prepared with a mass ratio of cement/quartz sand/water/high-performance water reducer of 1:2:0.4:0.005. The weighed cement was added to the mortar mixer, followed by the addition of water. The mortar mixer was rotated at a speed of 60 ± 5 r/min for 1 min. Quartz sand was then added, and the mixture was stirred at the same speed for 2 min. Subsequently, the mortar was poured into molds of different sizes. The molds were placed on a vibrating table and vibrated for 1 min. Finally, after 24 h of rest at a temperature of 20 ± 5 °C, the specimens were demolded and placed in a standard curing tank at a temperature of 20 ± 3 °C and a relative humidity of over 95% for 28 days of curing.

## 3. Results and Discussion

### 3.1. Infrared Spectroscopy Analysis (FTIR)

**Figure 4:**
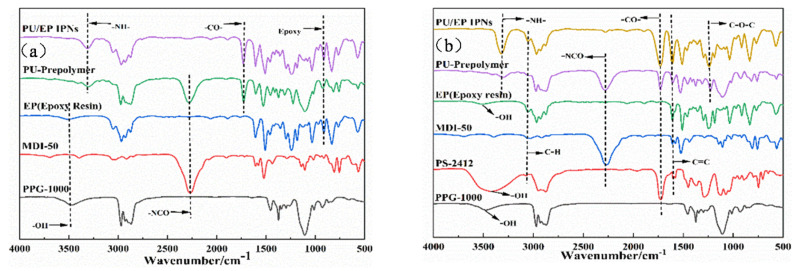
FT-IR spectra of (**a**) PG-1000, MDI-50, EP, PU-prepolymer, and polyether-based PU/EP IPNs; (**b**) PU-prepolymer and polyether-polyester-based PU/EP IPNs.

As shown in Figure 4b, the peak at 3500 cm^−1^ in the Figure represents the stretching vibration of hydroxyl groups in PPG-1000, PS-2412, and epoxy resin E51; the peak at 3380 cm^−1^ belongs to the stretching vibration of -NH- in PU prepolymer and PU/EP IPNs [24]. The peak at 2270 cm^−1^ is the stretching vibration absorption peak of -NCO in MDI-50 and the prepolymer of PU [25]. The absorption peak at 1700 cm^−1^ is attributed to the stretching vibrations of -CO- in PS-2412, the prepolymer of PU, and the IPNs of PU/EP. From the position and peak intensity of the characteristic peaks in Figure 4a,b, the -NHCOO- bond has been formed, and the -NCO in the polyurethane prepolymer has reacted with the hydroxyl group in the epoxy resin E51 [26]. The absorption peak of C–H stretching vibration on the benzene ring at a wave number of 3100 cm^−1^ is due to the introduction of PS-2412. The absorption peak for the asymmetric stretching vibration of the C=C in the benzene ring skeleton is at about 1600 cm^−1^, and the peak intensity is high, resulting in an increase in strength and a decrease in toughness of PU/EP IPNs.

### 3.2. Thermal Stability Analysis

**Figure 5:**
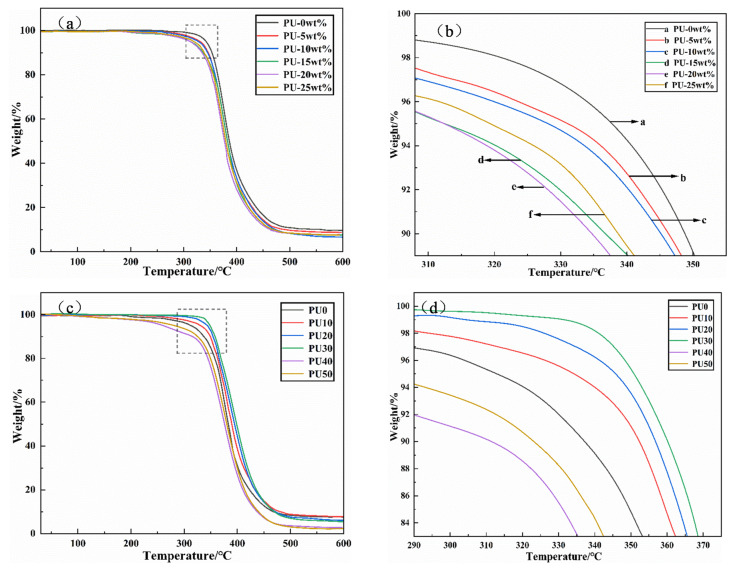
(**a**) Thermogravimetric analysis curves of epoxy resin and polyether-type PU/EP IPNs; (**b**) magnified part of thermogravimetric analysis curves of epoxy resin and polyether-type PU/EP IPNs; (**c**) thermogravimetric analysis curves of polyether-polyester type PU/EP IPNs at different ratios of PS-2412/PPG-1000; (**d**) magnified part of Thermogravimetric analysis curves of polyether-polyester type PU/EP IPNs at different ratios of PS-2412/PPG-1000.

As shown in Figure 5a,c, the weight loss of the PU/EP IPNs and the epoxy resin was roughly divided into two stages. The degradation in the first temperature stage (I) was mainly due to the thermal decomposition of the PU prepolymer, which mainly occurs in the range of 30–350 °C. The soft segments of the PU were more thermally stable than the hard segments, and the thermal degradation begins with the breaking of the C–N bonds in the amine carbonate and the hydrogen bonds between the soft and hard segments [27]. The second temperature stage (II) ranges from 350 to 600 °C. Within the range of 350–480 °C, it was related to the degradation of the soft segments of the PU prepolymer and the partial destruction of the epoxy resin main chain. At this time, the main chain of PU prepolymer and the interpenetrating network structure have basically been destroyed. When the temperature was between 480 and 600 °C, the main chain of the epoxy resin was completely destroyed.

As shown in Figure 5d, as the proportion of PS-2412 increases, the initial degradation temperature of PU/EP IPNs first increases and then decreases. The ester bond and benzene ring, with strong heat resistance in PS-2412, determined the increase in initial degradation temperature [28]. Amado [29] also confirmed that the thermal stability of polyester diols is higher than that of polyether diols.

As shown in Figure 5d, the thermal stability of PU30 and PU40 was poorer than that of PU0. The reason is the increase in the amount of PS-2412 added, and under the condition of excessive polyester soft segments. At this time, the migration at the junction of soft and hard segments is restricted, and the PU prepolymer formed is unstable at high temperatures and is more likely to dissociate, resulting in a decrease in the thermal stability of the PU/EP IPNs [30,31].

### 3.3. Scanning Electron Microscopy (SEM) Characterization

**Figure 6:**
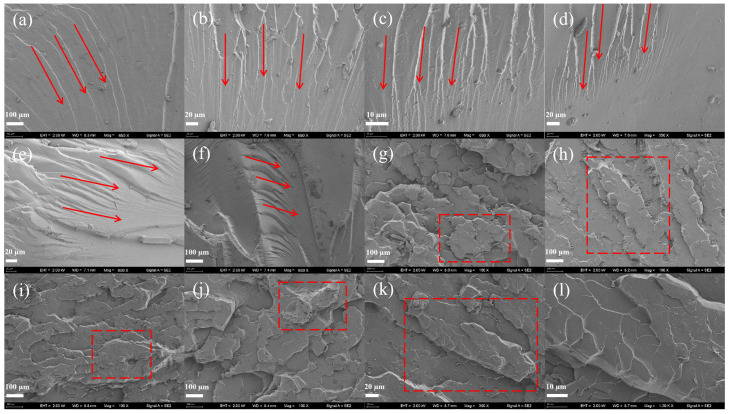
PU/EP IPNs fracture surface morphology (The red boxes and arrows highlight the features discussed in the text). (**a**): PU-0%; (**b**): PU-5%; (**c**): PU-10%; (**d**): PU-15%; (**e**): PU-20%; (**f**): PU-25%; (**g**): PU10; (**h**): PU20; (**i**): PU30; (**j**): PU40; (**k**,**l**): PU50.

The SEM images of the tensile fracture surfaces (Figure 6) were studied to study the fracture mechanism. As shown in Figure 6a–d, with the increased amount of PU prepolymer, the heterogeneity of the fracture surface increased. With crack patterns resembling “rivers” [32]. There is a clear phenomenon of crack branching with silver lines, which indicates that addition of PU prepolymer the fracture surface morphology of the epoxy resin matrix was changed significantly by addition of PU prepolymer, thereby enhancing its fracture toughness and achieving good toughening effects [33]. From Figure 6e,f, it was found that the shape of the cracks began to increase in size, and the number of cracks decreased. The phenomenon of silver lines also gradually decreased. This is because the PU prepolymer with longer flexible chain segments, being combined with the epoxy resin, led to excessive cross-linking and microphase separation, which resulted in different fracture toughness mechanisms [34].

As can be seen from Figure 6g–k, with the addition of the polyester diol PS-2412, the fracture surfaces of the composites exhibited a “scaly” texture, and with the proportion of PS-2412 increased, the texture was peeled and torn, which indicated that the increased content of PS-2412 enhanced the cross-linking degree of the PU/EP IPNs. There was a stronger interaction between the “hard segments” and “soft segments” of PU prepolymer molecular chains, and the reaction between the isocyanate groups and the hydroxyl groups of epoxy resin was maximized. From Figure 6k, it can be seen that the “scaly” texture gradually decreased and the texture was more concentrated on the fracture lines, which was due to the change in the PU prepolymer molecular structure, which increased the molecule entanglement in the PU/EP IPNs. Excessive participation of polyester diols in the reaction leads to uneven stress distribution in the interpenetrating network structure and phase segregation on the microscopic level, which is manifested as a concentrated distribution of line textures.

### 3.4. Mechanical Properties Analysis

**Figure 7:**
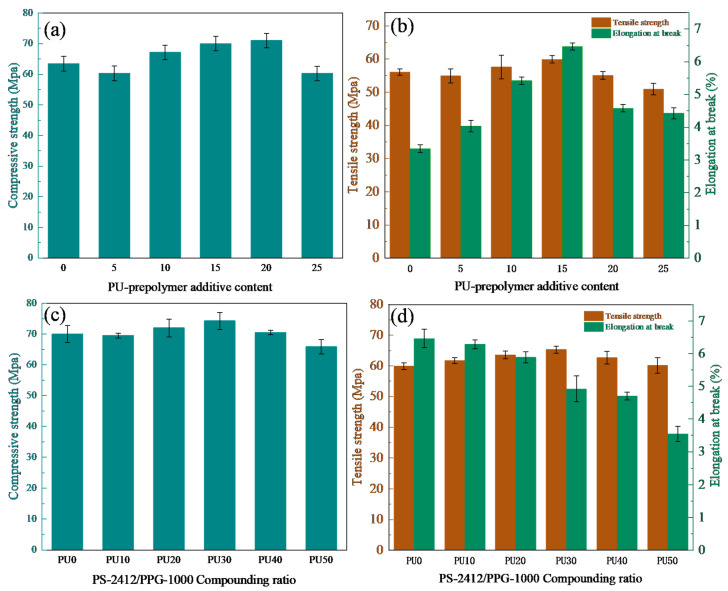
(**a**,**b**) Mechanical properties of polyether-based PU/EP IPNs; (**c**,**d**) mechanical properties of polyether-polyester-based PU/EP IPNs.

From Figure 7a,b, it can be seen that with the increased amount of PU prepolymer, the overall trend of the tensile and compressive strengths initially increases and then decreases. When the PU prepolymer content is 15 wt%, the compressive strength, tensile strength, and elongation at break of PU/EP IPNs reach their peak values of 69.99 MPa, 59.90 MPa, and 6.46%, respectively, which are 10.3%, 6.8%, and 92.8% higher than those of epoxy resin castings. With the increased amount of PU prepolymer, the PU prepolymer and epoxy resin formed a more stable and saturated interpenetrating polymer network (IPN). Therefore, a stronger stress/energy is required to wreck this interpenetrating network structure [5]. When the content of PU prepolymer exceeds 15 wt%, the cross-linking density of the interpenetrating network between the PU prepolymer and epoxy resin shifts from a stable saturated state to a supersaturated state. This leads to a decrease in compatibility between the two polymers and results in the uneven distribution of phases in the resin system and the reduction in the synergistic effect of the network between PU prepolymer and epoxy resin.

It can be seen from Figure 7c,d that the tensile strength and compressive strength initially increase with the addition of PS-2412. When PS-2412/PPG-1000 was 30/70, compared with the case without PS-2412 added, the elongation at break decreased by 23.8%, and the tensile strength and compressive strength increased by 11.4% and 6.07%, respectively. This was mainly because the addition of PS-2412 improved the cross-link density of the PU/EP IPNs, and diethylene glycol phthalic anhydride polymer diol contained a benzene ring structure, which increased the rigidity of the “soft segments” in the PU-prepolymer, thereby reducing the flexibility and toughness of the molecular chains in the PU/EP IPNs, and thus decreased the elongation at break of the PU/EP IPNs. In addition, the cohesive energy of the molecular chains with benzene rings and ester bonds was higher than that of the ether bonds, which increased the cohesive strength of the PU/EP IPNs. However, as the proportion of PS-2412 in the blend increased further, the cross-link density of the PU/EP IPNs became too high, and the molecular chain segments in the network could not uniformly bear the corresponding stress, causing the stress to concentrate on local network chains. This reduced the number of effective molecular network chains and increased the unevenness of the stress bearing [35]. Therefore, the combined effect of high cross-link density and low number of effective network chains led to an increase in brittleness of the PU/EP IPNs, a decrease in tensile strength, compressive strength, and a further reduction in elongation at break [36,37].

### 3.5. Bonding Strength Tests Analysis

**Figure 8:**
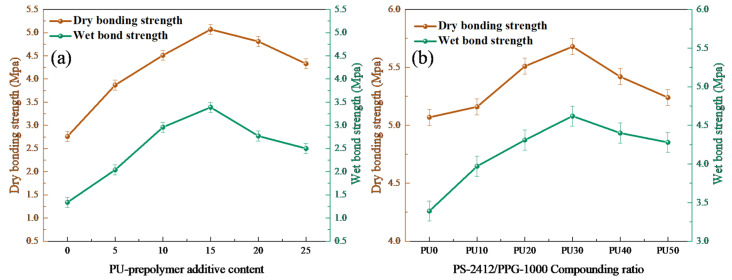
Adhesive properties of (**a**) polyether-based PU/EP IPNs; (**b**) polyether-polyester-based PU/EP IPNs.

As can be observed from Figure 8a, with the increased amount of PU-prepolymer addition, the dry and wet bonding strengths of the IPNs increased initially and then decreased. When the addition amount of PU prepolymer was 15 wt% of the epoxy resin, the dry and wet bond strengths of the PU/EP IPNs reached peak values of 5.07 MPa and 3.39 MPa, respectively, increasing by 83.6% and 127.5% compared to those of the epoxy resin. This was because the increase in PU-prepolymer continuously reacted with the epoxy resin to form a network structure, enabling it to withstand and disperse greater external stress, thereby enhancing the bonding strength between the PU/EP IPNs and concrete [38]. However, with further increase in the amount of PU-prepolymer, the PU/EP IPNs were affected by incompatibility and microphase separation caused by excessive saturation of both components, resulting in a slight decrease in the strength of the material itself, and subsequently a continuous decrease in the bonding strength between the PU/EP IPNs and concrete.

As shown in Figure 8b, with the increased proportion of the diethylene glycol phthalic anhydride polymer (PS-2412), the dry bonding strength and wet bonding strength of the PU/EP IPNs initially increase and then gradually decrease. When the PS-2412/PPG-1000 ratio was 30/70, the dry and wet bond strengths increased by 12.1% and 36.3%, respectively. The gradual increase was due to the fact that the addition of PS-2412 further increased the cross-link density of the PU/EP IPNs and enhanced their adhesive properties. The failure mode changed from interfacial failure to partial cohesive failure [38]. When the proportion of PS-2412 in the diol increased further, the excessive cross-link density in the PU/EP IPNs significantly increased the viscosity of the system, resulting in poor wetting properties and a significant decrease in cohesive strength of the PU/EP IPNs. This led to uneven stress bearing and increased brittleness, thereby gradually decreasing mechanical strength and adhesion.

### 3.6. Freeze–Thaw Tests Analysis

**Figure 9:**
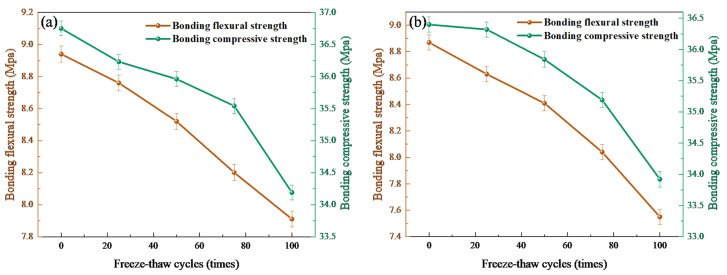
Bond flexural strength and bond compressive strength of concrete after freeze–thaw cycles: (**a**) polyether-type PU/EP IPNs; (**b**) polyether-polyester-type PU/EP IPNs repaired concrete.

**Figure 10:**
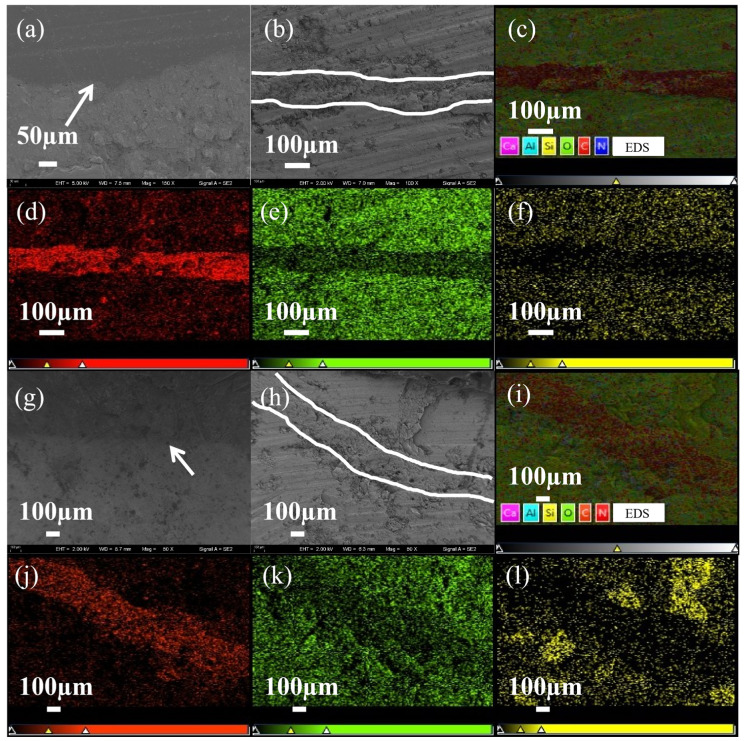
Comparison of SEM and EDS of repaired bond interface before and after freeze–thaw cycles. (**a**–**f**): polyether-type PU/EP IPNs repaired concrete; (**g**–**l**): polyether-polyester-type PU/EP IPNs repaired concrete; (**a**,**g**): SEM image of repaired interface before freeze–thaw cycle; (**b**,**h**): SEM image of repaired interface after 100 times of freeze–thaw cycle; (**c**,**i**): EDS electron image of repaired interface after 100 times of freeze–thaw cycle; (**d**,**j**): for C elemental labeling; (**e**,**k**): for O element labeling; (**f**,**l**): for Si element labeling.

**Figure 11:**
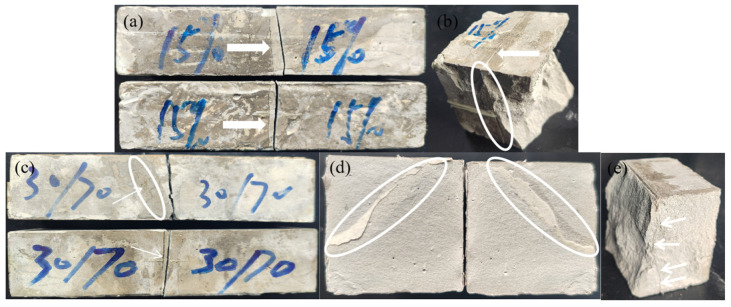
Concrete damage pattern after 100 freeze–thaw cycles: (**a**,**b**) Polyether PU/EP IPN repaired concrete, and (**c**–**e**) polyether-polyester PU/EP IPN repaired concrete.

As shown in Figure 9a,b, there was a much greater reduction of flexural and compressive bonding strengths in the later stages of freeze–thaw cycles. After repairing with polyether PU/EP IPNs, the bond flexural strength and bond compressive strength of concrete after freeze–thaw cycles decreased by 11.52% and 7.48%, respectively. After repairing with polyether-polyester PU/EP IPNs, the bond flexural strength and bond compressive strength of concrete after the 100th freeze–thaw cycle decreased by 14.88% and 6.81%, respectively. This was because the addition of ester groups reduces the freeze–thaw hydrolysis resistance of the repair material, making the repair interface more susceptible to damage, resulting in the formation of voids and microcracks at the interface. These defects will gradually propagate to the depths of the repair interface and the interior of the concrete substrate [39]. Therefore, the degradation of the microstructure on the interface reduced the interface strength. The SEM and EDS analysis of the repaired bonding interface before and after freeze–thaw cycles in Figure 10 also confirms the above viewpoint. Figure 11 shows the failure modes of concrete bonding flexural and compressive strength after 100 freeze–thaw cycles. It can be seen that the failure mode near the edge of the specimen is a mixed failure of bonding and substrate damage. The characteristic is that the damage occurs on the interface of the repaired concrete material, and there is a relatively loose pore structure in the transition zone, which leads to the formation of microcracks. The pore structure in the effective stress area of the repaired material concrete interface is relatively dense, and 100 freeze–thaw cycles could not damage it. The SEM results in Figure 10g,h confirmed the above viewpoint. Therefore, PU/EP IPNs repair materials of polyether/polyester type are more susceptible to freeze–thaw degradation, and with the increase in the number of freeze–thaw cycles, the durability and strength of the repaired concrete deteriorate irreversibly under the action of microcracks and freezing expansion, allowing more moisture to penetrate into the interface, exacerbating the degradation of interface performance.

As shown in Table 2, the material in this study demonstrates superior tensile strength compared to most existing systems, while maintaining moderate elongation at break and compressive strength. This indicates that our material design achieves a good balance between strength and toughness, thereby addressing the common limitation of existing materials which often exhibit extreme performance in only one aspect.

## 4. Conclusions

This study developed a series of polyurethane/epoxy resin interpenetrating polymer networks (PU/EP IPNs) with tunable properties by varying the ratio of phthalic anhydride polyester polyol (PS-2412) to polyether polyol (PPG-1000) in the soft segment. The key finding is that a PS-2412/PPG-1000 ratio of 30:70 yields an optimal crosslinking density, leading to a balanced enhancement in mechanical and thermal properties. Specifically, this formulation increased the tensile, compressive, dry bond, and wet bond strengths by 11.4%, 6.07%, 12.1%, and 36.3%, respectively, while maintaining a practically useful elongation at break and achieving the highest initial thermal degradation temperature. The performance improvement is attributed to the more uniform network structure and enhanced stress distribution enabled by the incorporated PS-2412. This work thus provides a viable strategy for toughening epoxy resins through synergistic soft-segment design. Future research should focus on scaling up the synthesis, evaluating long-term durability under real environmental conditions, and further tailoring the network architecture for specialized applications.

## Figures and Tables

**Table 1:** Ratios of diethylene glycol phthalic anhydride polymer and polyether polyol.

Numbering	PU0	PU10	PU20	PU30	PU40	PU50
PS-2412/PPG-1000	0/100	10/90	20/80	30/70	40/60	50/50

**Table 2:** Data comparison with other studies.

Materials	Tensile Strength	Elongation at Break	Compressive Strength
IPN-50/50 [16]	13.9 MPa	274.1%	
PU-EP = 2–8 [40]	25.0 MPa		110.0 MPa
85E [41]	100.2 MPa	4.64%	
This work	66.74 MPa	4.92%	77.24 MPa

## Data Availability

The data are presented in the tables and figures within the paper.
